# Diagnostic Criteria for Metabolic Syndrome in High-Altitude Regions: A Systematic Review

**DOI:** 10.3390/medicina58030451

**Published:** 2022-03-21

**Authors:** Claudia Beatriz Villegas-Abrill, Rubén Vidal-Espinoza, Rossana Gomez-Campos, Vladimiro Ibañez-Quispe, Charles Mendoza-Mollocondo, Sara Ruth Cuentas-Yupanqui, José Fuentes-López, Camilo Urra-Albornoz, Marco Cossio-Bolaños

**Affiliations:** 1 Instituto de Investigación en Ciencias de la Educación (IICE), Universidad Nacional del Altiplano de Puno, Puno 21001, Peru; claudiavillegas@unap.edu.pe (C.B.V.-A.); vibanez@unap.edu.pe (V.I.-Q.); cmmendoza@unap.edu.pe (C.M.-M.); jdfuentes@unap.edu.pe (J.F.-L.); 2 Escuela de Nutrición Humana, Facultad de Ciencias de la Salud, Universidad Nacional del Altiplano de Puno, Puno 21001, Peru; scuenta@unap.edu.pe; 3 Facultad de Educación, Universidad Católica Silva Henriquez, Santiago 8330225, Chile; rvidal@gmail.com; 4 Departamento de Diversidad e Inclusividad Educativa, Universidad Católica del Maule, Talca 3466706, Chile; 5 Escuela de Ciencias del Deporte y Actividad Física, Facultad de Salud, Universidad Santo Tomas, Santiago 8370003, Chile; c.urra.albornoz@gmail.com; 6 Departamento de Ciencias de la Actividad Física, Universidad Católica del Maule, Talca 3466706, Chile; mcossio1972@hotmail.com; 7 Centro de Investigación CINEMAROS SAC, Arequipa 04001, Peru

**Keywords:** metabolic syndrome, altitude, adults

## Abstract

*Background and Objectives*: Metabolic syndrome (MS) has many risk factors that are important to investigate in populations living at sea level and in high-altitude geographic regions. The aim was to identify the components of MS that cross-sectional studies use to assess in adult populations residing in high-altitude regions. *Materials and Methods*: A systematic review study was conducted. The Pubmed database was used. The search for original articles (cross-sectional) was performed from January 2013 to December 2020. The procedure was carried out by two researchers. The keywords used were metabolic syndrome, adults, and altitude regions. The search strategy considered the components of the PICOS tool. *Results:* Ten cross-sectional studies were identified in the Pubmed database from 2014 to 2020. Altitude levels varied between countries and regions, from 2060 to 4900 m above sea level. Three studies were conducted in both China and Peru, two studies in Ecuador, and one in Bolivia and India. The age ranges studied were from 18 to ~80 years of age, approximately. The components used to assess MS in most studies (between 9 to 10 studies) were body mass index (BMI), waist circumference (WC), blood pressure (BP), triglycerides (TG), high-density lipoproteins (HDL) and serum glucose (SG). *Conclusions*: This systematic review verified that the most commonly used domains in adult populations in various moderate- and high-altitude regions of the world are BMI, WC, BP, TG, HDL, and SG. These results suggest that in order to evaluate and/or investigate MS in subjects residing in high-altitude populations, at least four diagnostic domains should be considered in their protocols.

## 1. Introduction

Metabolic syndrome (MS) is a complex pathophysiological state that originates primarily from an imbalance in calorie intake and energy expenditure but is also affected by the genetic/epigenetic makeup of the individual, the predominance of sedentary lifestyle over physical activity, and other factors such as food quality and composition and gut microbiome composition [1].

Its components comprise abdominal obesity, insulin resistance (elevated fasting glucose), hypertension, and dyslipidemia (elevated triglyceride levels and decreased high-density lipoprotein (HDL) cholesterol levels) [2,3].

These abnormalities are closely related to increased risk of cardiovascular disease, type 2 diabetes and premature death [4,5,6,7].

In fact, studies in general have shown an increase in prevalence over time as a result of changes in diet, lifestyle, low physical activity, consumption of various processed foods, rich in saturated fats and sugar, as well as smoking, among other factors [8,9,10].

Prevalence figures in various populations around the world have reached 27.4% to 39% [11,12,13,14], which is evidence that MS like other chronic diseases, is a complex disease that depends on genetic and environmental factors [15,16].

In general, differences in genetic background, dietary habits, physical activity levels, age structure, sex, and levels of overnutrition and malnutrition can influence the prevalence of both metabolic syndrome and each of its components [17].

In this context, as MS is considered as a set of conditions that occur together and have a multifactorial etiology [18] and due to its large variations in climates, geography, lifestyles and ethnicities, it is important to investigate these risk factors not only in populations living at sea level, but also in geographic regions of altitude. 

In fact, several studies have reported that environment and altitude may also affect the incidence of MS, because people living in these regions tend to be thinner compared to those living in low altitude areas. This inverse association is independent of lifestyle, ethnicity and income [19].

On the other hand, it is also postulated that people living in high-altitude regions generate a favorable increase in cardiovascular conditions and respiratory efficiency, despite lower oxygen availability [20], and it is even highlighted that people living at altitude possess lower LDL (low-density lipoprotein), higher HDL, and better fasting glucose levels, as well as reduced obesity, compared to sea-level residents [20,21].

Therefore, this information could generate some advantages in the identification of prevalence in people living in high-altitude regions.

In fact, given that MS defines the coexistence of at least three of the five cardiometabolic risk factors (abdominal adiposity, hyperglycemia, hypertriglyceridemia, high-density lipoprotein cholesterol (HDL-C), and hypertension) [22], this study assumes that the primary studies performed in altitude regions at least consider three risk factors in their protocols

This information could help professionals and researchers working in the health sciences to adopt assessment protocols in the diagnosis and follow-up of MS and even to establish appropriate cut-off points for populations living in high-altitude regions in the future.

Therefore, the aim of the study was to identify the components of MS that cross-sectional studies use to assess in adult populations residing in high-altitude regions.

## 2. Materials and Methods

### 2.1. Type of Study

We conducted a systematic review study on cross-sectional research that has been conducted on metabolic syndrome in adults living in high-altitude geographic regions. To ensure the process of organization and systematized information we relied on the Preferred Reporting Items for Systematic Reviews and Meta-Analyses (PRISMA) statement [23]. The PubMed database of the US National Library of Medicine was used for the information search process.

### 2.2. Eligibility Criteria

To achieve the relevance of this systematic review, the original articles (cross-sectional) included the following keywords (1) metabolic syndrome; (2) adults; (3) altitude regions. Subsequently such words were grouped into two or three sets of words, and a new search was performed, such as, for example, metabolic syndrome and adults, metabolic syndrome and altitude regions. Matching and combination strategies were also used to search for research, whose terms were searched for in the study titles, abstract and keywords. 

Studies that included systematic reviews, bibliographic reviews and letters to the editor were excluded from the analysis. Therefore, this process was limited to original articles (cross-sectional) that investigated adults aged 18 years and older up to ~80 years of age. Original articles were also excluded if they met the eligibility criteria but were not accessible in the full version (not available electronically or in hard copy or requested from the authors but not submitted).

### 2.3. Search Strategy

The electronic search covered a period of one month (January 2021). Specific searches in the most-cited journals and authors and in the reference lists of the articles ensured the location of all relevant articles. The basis for the search strategy was considered using the components of the PICOS (Population, Interventions, Comparators, Outcomes, and Study design) tool, these being P: adults ≥ 18 years who participated as a sample in metabolic syndrome topics; I: components of MS; C: present different components of the evaluated MS; O: describe methodological characteristics of the investigated sample (number, age, altitude level, city) in studies evaluating MS in altitude populations; and S: cross-sectional studies. 

### 2.4. Methodological Quality

The assessment of methodological quality was performed independently by two reviewers (MC, RG), who analyzed the selected studies of this systematic review and resolved disagreements in the consensus analysis. The methodological quality review was performed considering the 11 items recommended by the Agency for Healthcare Research and Quality (AHRQ). Each item has a score of “Yes”, “No”, or “Unclean” to judge in the systematized study.

The following items were considered: Define the source of information (survey, record review); list the inclusion and exclusion criteria for exposed subjects or refer to previous publications; indicate the time period used to identify patients; indicate whether subjects were consecutive or not, if not population-based; indicate whether the assessors of the subjective components of the study were masked to the participants’ status; describe any assessments performed for quality assurance purposes (e.g., testing/re-testing of primary outcome measurements); explain any exclusions of patients from analyses. Describe any assessments conducted for quality assurance purposes (e.g., test/re-retest of primary outcome measurements); explain any exclusions of patients from analysis; describe how confounders were assessed and/or controlled for; if applicable, explain how missing data were handled in the analysis; summarize patient response rates and completeness of data collection; clarify what follow-up was expected, if any, and the percentage of patients for whom incomplete data or follow-up was obtained. 

### 2.5. Data Extraction and Analysis

The data extraction process for each article was carried out using a structured script (observation sheet) that included the following points: authors, (age range and sex of study participants), altitude (city and country), and components of metabolic syndrome used.

A first reviewer performed the data extraction described above, and the second reviewer checked the data extraction. This ensured concordance of the data.

For the presentation of the results in terms of systematization, descriptive analysis (ranges and frequencies) was used to quantify the components of the metabolic syndrome used in altitude regions.

## 3. Results

**Figure 1:**
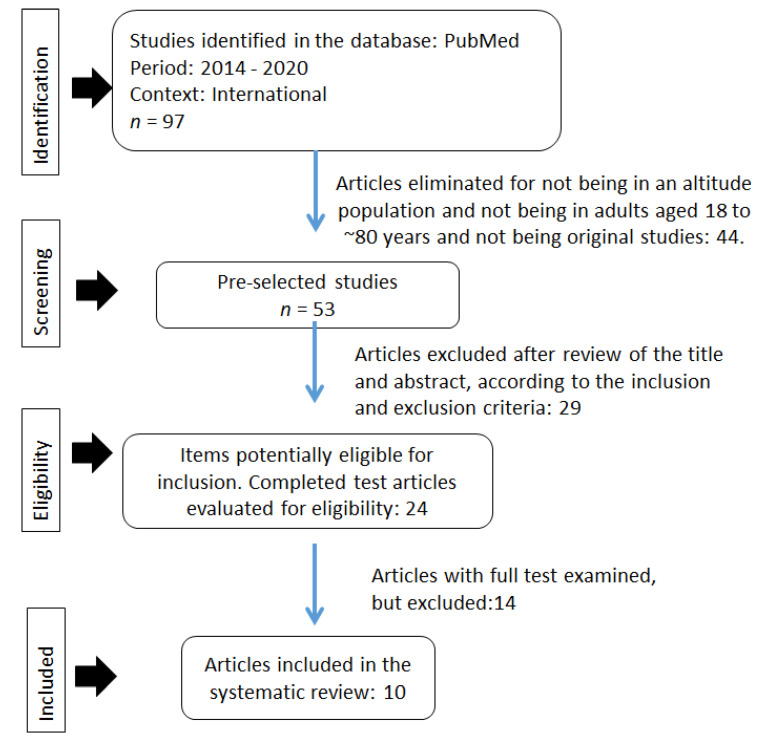
PRISMA flow chart for the systematization of original articles 2014–2020.

Figure 1 shows the PRISMA flow chart showing the study selection process developed. A total of 97 studies were identified worldwide, which were considered as potential studies for systematization. Subsequently, 44 studies were eliminated as they were not original studies in high-altitude populations. In the next stage, the titles and abstracts were read, considering the inclusion and exclusion criteria, and 29 articles were eliminated. In the third stage, of the 24 eligible studies, which were read in their entirety, 14 that were not cross-sectional studies were eliminated. Finally, 10 articles were considered in this review.

The results of the quality analysis of the articles are shown in Table 1. All studies included in this systematic review define the sources of information, presented inclusion and exclusion criteria for participants, present patient responses. Fifty percent indicated the time period used to identify the patients, only 30% clearly indicated the management of confidentiality of the results.

In relation to the control of the measures, only 10% of the studies indicated that they were controlled. Likewise, two studies (20%) described the assessment and control of confounding factors, and only one study explained how missing data were treated.

In general, the evaluation of methodological quality showed that the two studies with the highest scores were Sherpa et al. [10] and Huang et al. [24] (9 and 8 points, respectively), followed by Guo et al. [25], Lin et al. [26], Hurtado-Arestegui et al. [27] and Herrera-Enriquez, Narvaez Guerra [28] (with 7 and 6 points, respectively); and Lopez-Pascual et al. [29], De Ferrari et al. [30], and Miele et al. [7] (5 points) on this scale.

The indicators that characterize the cross-sectional studies can be seen in Table 2. Ten studies published from 2014 to 2020 were systematized; in addition, seven journals were identified that published cross-sectional research related to MS in high-altitude regions.

Table 3 describes the sample size used in the studies, as well as the age range, city (country), and altitude level where the studies were conducted. Of the ten systematized studies, three were conducted in China and Peru, two in Ecuador, and one each in Bolivia and India. The ages that have been investigated vary among the studies, for example, seven studies have included subjects from 30 to ~80 years of age and three studies from 18, 20, and 27 years of age onwards, up to 48, 60, and 70 years of age approximately. 

The altitude levels of each geographic region also vary between countries, e.g., the altitude levels can be seen in Table 3 and Table 4. The studies in general were carried out in China, Derong (2060 m), Sichuan (3200 m) and Tibet (4900 m), in Ecuador, Imbadura (2500 m), Quito (2850 m) and Riobamba (2754 m), in India, Ladakn (3505 m), in Bolivia, La Paz (3640 m) and Peru, in Chivay (3635 m) and Puno (3825 m).

**Figure 2:**
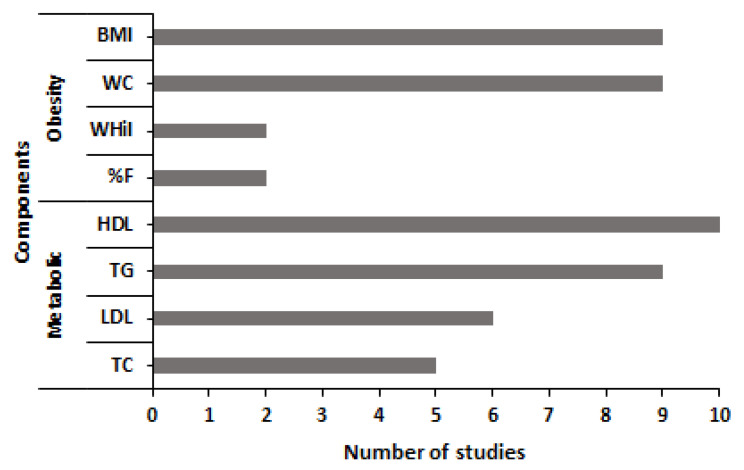
Components of the metabolic syndrome used to assess adults living in high-altitude regions (BMI: Body Mass Index, WHiI: Waist hip index, WC: waist circumference, WHeI: waist height index, BP: blood pressure, SG: serum glucose, TC: total cholesterol, TG: triglycerides, %F: body fat percentage; HDL: high-density lipoprotein, LDL: low-density lipoprotein).

Of the ten systematized studies (Figure 2), it is observed that the majority (between 9 and 10 studies) have evaluated BP, obesity indicators (BMI and WC), HDL, TG and SG. However, 02 studies have considered waist-hip index (WHiI) and body fat percentage (%F) (measured using bioelectrical impedance analysis) as indicators of obesity. In addition, six studies have considered LDL, and five studies have considered total cholesterol (TC). In general, BMI, WC, BP, TG, HDL and SG are those frequently used in samples of adults living at moderate and high altitudes in China, India, Ecuador, Bolivia, and Peru, respectively.

## 4. Discussion

This review aimed to identify the components of MS that cross-sectional studies used in adult populations residing in high-altitude regions. The results reported ten cross-sectional studies that have been conducted in moderate- and high-altitude regions in China, India, Ecuador, Bolivia, and Peru.

In general, most of the investigations conducted at high altitude have used five components of MS, such as BP, obesity indicators (BMI and WC), HDL cholesterol, TG, and SG. These findings are consistent with reports proposed by the World Health Organization (WHO), the National Cholesterol Education Program [32], and by the International Diabetes Federation (IDF), which are usually considered to be the most popular and are generally used in surveys and health care plans in various populations around the world [22]. 

For example, the WHO [33] highlights four components such as: HDL cholesterol, TG, WHiH or BMI and BP; however, it considers as a priority the presence of insulin or glucose resistance, and together with it, two or more of the other parameters are added. 

The National Cholesterol Education Program [32] emphasizes the presence of three or more of the five parameters they propose. These include SG, HDL cholesterol, TG, WC, and BP. While the International Diabetes Federation [34] highlight in their report the presence of five parameters (SG, HDL cholesterol, TG, WC and BP), where WC turns out to be a primary parameter, and along with that anthropometric measure, two or more of the remaining three should be increased.

In essence, systematized cross-sectional studies have shown that in high-altitude regions, four of the five suggested components should be used. This information is relevant, since it can be used for the prevention and treatment of young people and adults living in high-altitude regions.

It is currently estimated that more than 400 million people worldwide reside in high-altitude areas above 1500 m above sea level (MASL) [24], representing a major physiological challenge for residents of these regions. 

In general, permanent residents exhibit physiological adaptations to environmental pressure and lower oxygen availability, such as elevated hemoglobin concentration, enlarged lung volume, and decreased hypoxic ventilatory response [21]. These physiological aspects of human adaptation at altitude have allowed for the development of multiple studies that have to do with cardiovascular and respiratory efficiency, lower LDL cholesterol values, higher HDL cholesterol, better fasting glucose levels, reduced obesity rates, compared to sea level residents, and they even usually present lower presence of diabetes and hypertension [20,21,31,35,36].

In general, genetic and physiological adaptations to hypoxia affect glucose metabolism and trigger appetite suppression and consequently reduced caloric intake [37]. These aspects could be indicators of the urgent need for the presence of proper criteria and cut-off points to assess MS at altitude. This is due to the fact that not only physiological reasons may be justified, but also lifestyle differences between regions may be determinant.

Therefore, future studies should be interested in proposing reference values for each of the five components that define the MS for residents of moderate and high altitudes, although it is necessary to take into consideration that the MS of non-Andean populations (for example, Tibetans) cannot be compared with those performed in inhabitants of the Andes, due to the differences in phenotypes and genotypes [38], in addition to considering the differences in lifestyle between regions [39].

Consequently, data and research on the components, reference values and cut-off points on SM in various altitude regions of the world still remain insipient, especially if we are talking about South American countries, so future studies should be interested in developing new proposals that allow more accurate assessment, diagnosis, and monitoring of altitude residents. This is the first study that reported on the components used in high-altitude regions, which opens new perspectives for researchers in high-altitude regions, since, as observed in this study, there is little information on the prevalence of MS in areas of moderate and high altitude in the world.

This study has some limitations that have to do with the use of a single database and the inclusion of cross-sectional studies, so other studies should be interested in expanding the search for information to other databases, as well as extending their search to longitudinal and experimental investigations to determine the effect of high altitude on metabolic syndrome. In addition, this systematic review is one of the first of its kind, so it can serve as a baseline for future studies and the systematized information can be useful, not only for researchers, but also for public health professionals working in high-altitude regions, and even for developing national strategies for the prevention and treatment of MS in regions of moderate and high altitude.

## 5. Conclusions

In conclusion, this study verified that the most commonly used domains in adult populations in various moderate and high-altitude regions of the world are BMI, WC, BP, TG, HDL and SG. These results suggest that in order to evaluate and/or investigate MS in subjects residing in high-altitude populations, at least 4 diagnostic domains should be considered in their protocols. This information could help to propose in the future specific domains for people living in high-altitude geographic regions of the world.

## Figures and Tables

**Table 1:** Analysis of the methodological quality of the articles selected for this systematic review.

No	Item	Huang et al. [24]	Lopez-Pascual et al. [29]	Sherpa et al. [10]	Lin et al. [26]	Hurtado-Arestegui et al. [27]	De Ferrari et al. [30]	Miele et al. [7]	Gou et al. [25]	Herrera-Enríquez, Narváez-Guerra [28]	Salazar-Lugo et al. [31]
1	Define the source of information (survey, record review).	Yes	Yes	Yes	Yes	Yes	Yes	Yes	Yes	Yes	Yes
2	List inclusion and exclusion criteria for exposed subjects or refer to previous publications	Yes	Yes	Yes	Yes	Yes	Yes	Yes	Yes	Yes	Yes
3	Indicate the time period used to identify patients	Yes	No	Yes	Yes	No	No	No	Yes	Yes	No
4	Indicate whether subjects were consecutive or not, if not population based.	Yes	Yes	Yes	Yes	Yes	Yes	Yes	Yes	Yes	Yes
5	Indicate whether the evaluators of the subjective components of the study were masked to the participants’ status	Yes	No	Yes	No	No	No	No	Yes	No	No
6	Describe any evaluations performed for quality assurance purposes (e.g., testing/repeat of primary outcome measurements).	No	No	No	No	Yes	No	No	No	No	No
7	Explain any exclusions of patients from analyses.	Yes	Yes	Yes	Yes	Yes	Yes	Yes	Yes	Yes	No
8	Describe how confounders were assessed and/or controlled for.	Yes	Unclear	Yes	No	No	Unclear	No	No	No	No
9	If applicable, explain how missing data were handled in the analysis.	No	No	Yes	No	No	No	No	No	No	No
10	Summarize patient response rates and completeness of data collection.	Yes	Yes	Yes	Yes	Yes	Yes	Yes	Yes	yes	Yes
11	Clarify what follow-up was expected, if any, and the percentage of patients for whom incomplete data or follow-up was obtained	No	No	No	No	No	No	No	No	No	No

**Table 2:** Indicators of the articles systematized for the study.

N°	Author(s)	Year	Title	Journal
1	Huang et al. [24].	2020	Metabolic syndrome in native populations living at high altitude: a cross-sectional survey in Derong, China.	BMJ Open.
2	Lopez-Pascual et al. [29].	2018	Inverse Association Between Metabolic Syndrome and Altitude: A Cross-Sectional Study in an Adult Population of Ecuador	Frontiers in Endocrinology, Clinical Diabetes.
3	Sherpa et al. [10].	2013	Prevalence of metabolic syndrome and common metabolic components in high-altitude farmers and herdsmen at 3700 m in Tibet.	High Altitude & Medicine Biology.
4	Lin et al. [26].	2018	The prevalence of obesity and metabolic syndrome in Tibetan immigrants living in high altitude areas in Ladakh, India.	Obesity Research & Clinical Practrice.
5	Hurtado-Arestegui et al. [27]	2018	Higher prevalence of unrecognized kidney disease at high altitude.	Journal of Nephrology.
6	De Ferrari et al. [30]	2014	Prevalence, clinical profile, iron status, and subject-specific traits for excessive erythrocytosis in Andean adults living permanently at 3825 m above sea level.	Chest Journal
7	Miele et al. [7]	2016	Increased Cardiometabolic Risk and Worsening Hypoxemia at High Altitude.	High Altitude & Medicine Biology.
8	Gou et al. [25]	2020	The Prevalence and Risk Factors of High-Altitude Pulmonary Hypertension Among Native Tibetans in Sichuan Province, China.	High Altitude & Medicine Biology.
9	Herrera-Enríquez, Narváez-Guerra [28]	2017	Discordance of metabolic syndrome and abdominal obesity prevalence according to different criteria in Andean highlanders: A community-based study.	Diabetes & Metabolic Syndrome: Clinical Research & Reviews
10	Salazar-Lugo et al. [31]	2016	Factores bioquímicos y nutricionales asociados a la viscosidad sanguínea en adultos de la sierra urbana (Imbabura), Ecuador.	Investigación Clínica

**Table 3:** Methodological characteristics of research conducted on metabolic syndrome in high-altitude regions.

N°	Author(s)	Samples	Ages (Year)	Altitude (m)	City (Country)
1	Huang et al. [24]	*n* = 5053 M = 2221 W = 2832	18–70	2060–3820	Derong (China)
2	Lopez-Pascual et al. [29]	*n* = 260 M = 168 W = 92	27–48	2758–2787	Guayaquil, Triunfo y Riobamba (Ecuador)
3	Sherpa et al. [10]	*n* = 692 M = 317 W = 375	30–80	3700	Lhunzhub y Qushu, Region del Tíbet (China)
4	Lin et al. [26]	*n* = 149 M = 47 W = 102	40–73	3505	Ladakh (India)
5	Hurtado-Arestegui et al. [27]	*n* = 293 M = 145 W = 148	40–60	3640–4500	La Paz (Bolivia)
6	De Ferrari et al. [30]	*n* = 1065 M = 518 W = 547	42–67	3825	Puno, Perú
7	Miele et al. [7]	*n* = 1065 M = 518 W = 547	42–67	3825	Puno, Perú
8	Gou et al. [25]	*n* = 1129 M = 440 W = 689	32–60	3200	Sichuan, China
9	Herrera-Enríquez, Narváez-Guerra [28]	*n* = 237 M = 110 W = 127	48–82	3635	Chivay, Perú
10	Salazar-Lugo et al. [31]	*n* = 237 M = 128 W = 109	20–60	2200	Imbabura (Ecuador)

Legend: *n*: sample size, M: men, W: women, m: meters.

**Table 4:** Altitude level of cities and countries that have investigated metabolic syndrome in adults.

Country/City	Altitude (masl)
China	
Derong	2060
Tibet	4900
Sichuan	3200
Ecuador	
Riobamba	2754
Quito	2850
Imbabura	2500
India	
Ladakh	3505
Bolivia	
La Paz	3640
Perú	
Puno	3825
Chivay	3635

Legend: masl: meters above sea level.
